# The linear ANRIL transcript P14AS regulates the NF-κB signaling to promote colon cancer progression

**DOI:** 10.1186/s10020-023-00761-z

**Published:** 2023-12-01

**Authors:** Wanru Ma, Junhua Hu

**Affiliations:** Department of Blood Transfusion, Beijing Hospital, National Center of Gerontology, Institute of Geriatric Medicine, Chinese Academy of Medical Sciences, Beijing, 100730 P. R. China

**Keywords:** ANRIL, P14AS, miR-23a-5p, UBE2D3, NF-κB

## Abstract

**Background:**

The linear long non-coding RNA *P14AS* has previously been reported to be dysregulated in colon cancer, but the mechanistic role that *P14AS* plays in colon cancer progression has yet to be clarified. Accordingly, this study was developed to explore the regulatory functions of *ANRIL* linear transcript-*P14AS* in cancer.

**Methods:**

The expression of *P14AS*, *ANRIL*, *miR-23a-5p* and their target genes were detected by quantitative real-time polymerase chain reaction (qRT-PCR) and western blot. Cell supernatants of IL6 and IL8 were measured by Enzyme linked immunosorbent (ELISA) assay. Dual-luciferase reporter assays, RNA immunoprecipitation, or pull-down assays were used to confirm the target association between *miR-23a-5p* and *P14AS* or *UBE2D3*. Cell proliferation and chemosensitivity of NF-κB inhibitor BAY 11-7085 were evaluated by cell counting kit 8 (CCK8).

**Results:**

When *P14AS* was overexpressed in colon cancer cell lines, enhanced TNF-NF-κB signaling pathway activity was observed together with increases in *IL6* and *IL8* expression. The Pita, miRanda, and RNA hybrid databases revealed the ability of *miR-23a-5p* to interact with *P14AS*, while UBE2D3 was further identified as a *miR-23a-5p* target gene. The results of dual-luciferase reporter, RNA pull-down, and RNA immunoprecipitation experiments confirmed these direct interactions among *P14AS*/*miR-23a-5p*/UBE2D3. The degradation of IκBa mediated by UBE2D3 may contribute to enhanced NF-κB signaling in these cells. Moreover, the beneficial impact of *P14AS* on colon cancer cell growth was eliminated when cells were treated with *miR-23a-5p* inhibitors or UBE2D3 was silenced. As such, these findings strongly supported a role for the UBE2D3/IκBa/NF-κB signaling axis as a mediator of the ability of *P14AS* to promote colon cancer progression.

**Conclusions:**

These data suggested a mechanism through which the linear *ANRIL* transcript *P14AS* can promote inflammation and colon cancer progression through the sequestration of *miR-23a-5p* and the modulation of NF-κB signaling activity, thus highlighting *P14AS* as a promising target for therapeutic intervention efforts.

**Supplementary Information:**

The online version contains supplementary material available at 10.1186/s10020-023-00761-z.

## Background

The well-known *CDKN2A/B* locus is associated with human tumors and metabolic diseases. Although this locus has three famous tumor suppressor genes (*P16*^*INK4A*^, *P14*^*ARF*^, and *P15*^*INK4B*^), the antisense strand of this genetic locus encodes the long non-coding RNAs (lncRNAs) *ANRIL*, as well as *ANRIL’s* linear transcript, *P14AS*, the former of which is capable of binding the polycomb repressive complex-1/2 (PRC-1/2) to inhibit the expression of *P15*^*INK4B*^ and *P16*^*INK4*^. *P14AS* promotes *ANRIL* upregulation through binding to AUF1(Ma et al. 2020; Li et al. 2022). Colon cancer (CC) cells exhibit the overexpression of both *ANRIL* and *P14AS*, which promote tumor development and malignant cell proliferation in vitro and in vivo(Ma et al. 2020). MicroRNAs (miRNAs) are small 19–25 nucleotide transcripts that can regulate gene expression at both the transcriptional and post-transcriptional levels(Michlewski and Cáceres 2019; Ha and Kim 2014). Many miRNAs have been established as valuable biomarkers associated with various cancer types. Regulatory interactions between *ANRIL* and specific miRNAs have also been observed in many cancers, such as the interactions between *ANRIL* and both *miR-125a* and *let-7a* in CC and nasopharyngeal cancer(Hu et al. 2017; Wang et al. 2017; Zhang et al. 2018), or the *ANRIL-miR-99a/miR-449a* regulatory axis in gastric cancer(Zhang et al. 2014). However, whether miRNAs similarly interact with *P14AS* and how these interactions influence the pathogenesis of CC have yet to be established. Single nucleotide polymorphisms (SNPs) in the *ANRIL* sequence have been linked to various diseases including type 2 diabetes and coronary atherosclerosis(Kong et al. 2018). Since atherosclerosis develops due to chronic and progressive inflammation, *ANRIL* expression may be related to inflammation. In endothelial cells, NF-κB and TNFα can function as pro-inflammatory mediators that promote *ANRIL* upregulation and the expression of cytokines, including IL6 and IL8 through interactions with the transcription factor YY1 via the TNFα-NF-κB- *ANRIL*/YY1-IL6/8 signaling axis, thereby altering the local endothelial microenvironment(Zhou et al. 2016; Gupta et al. 2020). *ANRIL* can also drive tumor progression by regulating various pathways including the mTOR, MAPK, PI3K/AKT, and TGF-β(Yu et al. 2018; Wang et al. 2022a, b; Liu et al. 2019; Dong et al. 2018). An analysis of HCT116 cells in which *P14AS* was stably overexpressed revealed enrichment in KEGG pathways, including the TNF signaling pathway (Corrected *P* = 0.012). Further research is required to determine whether and how *P14AS* modulates the TNF-NF-κB signaling axis.

Here, *P14AS* was demonstrated to function as a molecular sponge that sequesters *miR-23a-5p* and promotes the upregulation of the *miR-23a-5p* target protein Ubiquitin Conjugating Enzyme E2 D3 (UBE2D3). Through interactions with the E2 CDC34 and the SCF(FBXW11) E3 ligase complex, UBE2D3 can promote NFκBIA (IκBa) polyubiquitination, thus driving its proteasome-mediated degradation. These results support a potential hypothesis wherein *P14AS* can promote tumorigenesis and disease progression via the *miR-23a-5p*/UBE2D3/IκBa pathway-mediated activation of NF-κB signaling activity.

## Materials and methods

### Cell culture

HCT116, SW480, LOVO, and HEK293T were provided as a kind gift from Professor Dajun Deng at Peking Union Medical College Hospital, and were cultured in DMEM (Corning, VA, USA) containing 10% fetal bovine serum (Gibco, Australia) and 1% penicillin/streptomycin (Gibco, NY, USA) in a 5% CO_2_ incubator at 37 °C.

### Cell transfection

The PCDH-CMV-EF1a-copGFP-T2A-Puro lentiviral vector was used to generate a *P14AS* expression construct by Syngentech Co., Ltd. (Beijing, China). The psiCHECK2 vector was obtained from Sangon Biotech Co., Ltd (Shanghai, China). Lipofectamine 3000 (Invitrogen, CA, USA) was used to transfect cells with appropriate miRNAs, siRNAs, or other constructs based on provided instructions. Colon cancer cells were transfected with miRNA mimics (final concentration 100nM) or miRNA inhibitors (final concentration 100nM) or siRNA (final concentration 100nM) for 48-72 h. Post-transfection, cells were collected for CCK8 assay or WB assay or qRT-PCR. Stably transfected HCT116, SW480, and LOVO cells were obtained through culture in media containing puromycin (1 µg/mL, InvivoGen, CA, USA).

### CCK-8 assays

A TransDetect Cell Counting Kit-8 (CCK-8, TransGen Biotech, Beijing, China) was used based on the provided directions to assess transfected cell viability. Briefly, 100 µL of media containing either 20,000 or 80,000 cells/mL (for proliferation and cytotoxicity assays, respectively) was added to each well of a 96-well plate. In cytotoxicity assays, after allowing cells to adhere to the plate, media was exchanged for media containing a range of BAY 11-7085 (Selleck, Shanghai, China) concentrations (0, 2, 4, 8, 12, 16, 32µM) or DMSO. At appropriate time points, CCK-8 solution (10 µL) was added per well, followed by an additional 3 h incubation at 37 °C. Absorbance was then measured at 450 and 630 nm with a BioRad microplate reader. Proliferation was assessed once daily on 5 sequential days, and average absorbance values were measured.

### RNA extract and qPCR

Trizol (TransGen Biotech, Beijing, China) was used to extract cellular RNA, after which a TransScript First-Strand cDNA Synthesis SuperMix (Roche, IN, USA) was used to prepare cDNA. Then, FastStart Universal SYBR Green Master (ROX) (TransGen Biotech, Beijing, China) was used to conduct qPCR analyses with an ABI-7500 Fast system (Applied Biosystems) with the following settings: 94 °C for 30 s; 40 cycles of 94 °C for 5 s, 60 °C for 15 s, 72 °C for 34 s. GAPDH was used as a normalization control. Primers used for this study are listed in Supplementary Table S1.

### Western blotting

NP-40 buffer (Solarbio, Beijing, China) supplemented with protease inhibitors (LabLead, Beijing, China) was used to extract protein from cells, and these samples were then separated via SDS-PAGE and transferred onto PVDF membranes (Merck Millipore). Following a 1 h room temperature blocking step using 5% skim milk, the membranes were probed with appropriate primary antibodies overnight at 4 °C followed by probing for 1 h at room temperature with secondary anti-mouse or anti-rabbit IgG (ZSGB Biotech, Beijing, China). A chemiluminescence detection system (Cytiva, Amersham ImageQuant 800, Japan) was then used for protein detection. The primary antibodies used were specific for AUF1 (Abcam, ab61193, UK), CDCP1 (Abcam, ab252947), ADAM10 (Abcam, ab124695), UBE2D3 (Abcam, ab176568), IκBa (Abcam, ab32518), Ago2 (Abcam, ab186733), YY1 (Santa Cruz, sc-7341, TX, USA), HNF3a (Santa Cruz, sc-514,695), GAPDH (Protein Tech, 50430-2-AP; China), β-tubulin (Abcam, ab6046). Antibodies were used at dilutions ranging from 1:1000-1:5000.

### RNA pull-down assay

Biotinylated *P14AS* probes (#1-#6) and corresponding control probes (#1-#2) that had been synthesized in vitro by RiboBio (Guangzhou, China) were incubated with SW480 cell lysates, after which an RNA pull-down assay was performed based on protocols published previously(Ma et al. 2020).

### RIP assay

An RNA-Binding Protein Immunoprecipitation Kit (Cat# 17–701, EZ Magna, Millipore, USA) was used based on the directions provided. Total RNA bound to AUF1 or Ago2 was precipitated and isolated with anti-AUF1 (Abcam, ab61193, Cambridge, UK) or anti-Ago2 (Abcam, ab186733).

### Luciferase reporter assay

HindIII was used to insert the full-length *P14AS* sequence containing wild-type or mutated binding sites for target miRNAs (miR-23a-5p, miR-6855-5p, and miR-6842-5p) into the pGL3-control vector (Promega, WI, USA). The 3’-UTR sequences for target genes or mutant isoforms were inserted into the psiCHECK2 vector using XhoIand NotI(Sangon Biotech, Shanghai, China). For further details regarding the binding sites used to generate these plasmids, see Fig. 3E. After adhering overnight to 12-well plates, cells were co-transfected with WT or mutated luciferase reporter vectors together with miRNA mimics (final concentration 100nM), inhibitors (final concentration 100nM), or control vectors and incubated for 48 h. A dual luciferase reporter assay kit (Promega, WI, USA) was used based on the directions provided.

### ELISA

Supernatants were collected from cells that had been stably transfected, and the concentrations IL-6 (EH004-96) and IL-8 (EH005-96) therein were measured using appropriate ELISA kits (ExCell, Shanghai, China) based on the manufacturer’s instructions.

### ChIP assay

A ChIP kit (Beyotime Biotech, Beijing, China) was used based on the provided directions, after which the fragments of DNA precipitated using YY1 (Santa Cruz, sc-7341, TX, USA), HNF3a (Santa Cruz, sc-514,695), and control IgG were analyzed via qPCR.

### RNA-seq and data analyses

The transcriptomes of the stably P14AS-overexpressed HCT116 cells were sequenced by RiboBio Co., Ltd. (Guangzhou, China). The data sets were deposited in the Gene Expression Omnibus (GEO) database with accession number GSE127905. KEGG pathway analysis for differentially expression genes was performed using KOBAS3.0 software (http://www.genome.jp/kegg). P14AS-binding miRNAs and miRNA-target proteins were analyzed using the miRanda, Pita, RNAhybrid, and TarPmiR databases.

### Statistical analyses

Data were compared using two-sample Student’s t-tests and presented using GraphPad Prism 7.0 software (Dotmaticus, USA). Results are reported as means ± SD. Pearson correlation analyses were used to assess relationships between variables. Two-sided statistical tests were used for all analyses. *P < 0.05, **P < 0.01, *N.S*: not significant.

## Results

### *P14AS* positively regulates TNF signaling in HCT116 cells

**Fig. 1:**
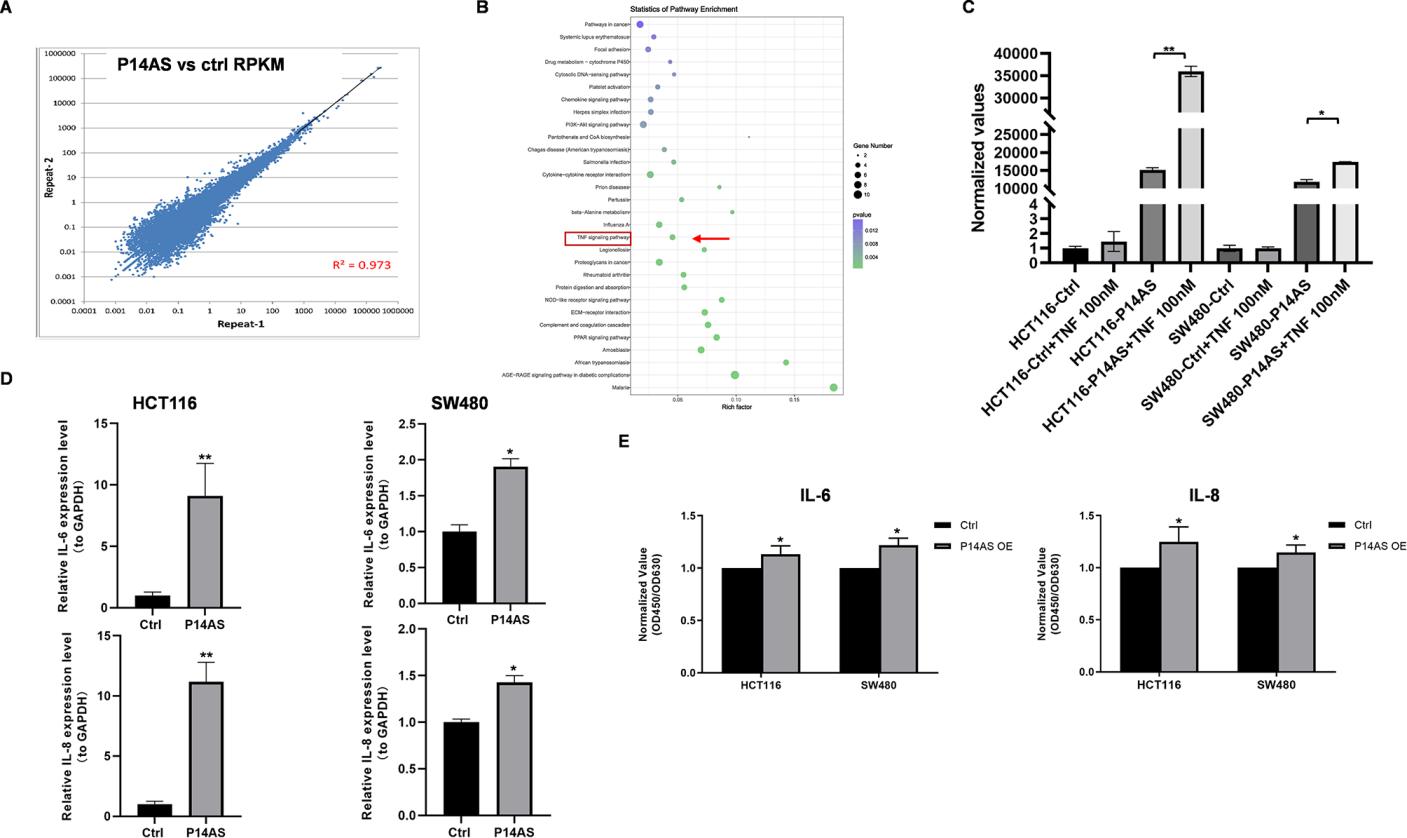
TNF-NF-κB-IL-6/IL-8 signaling is regulated by *P14AS* in CC cells. **(A)** High correlation of gene expression between two biological repeats for RNA-seq. **(B)** TNF signaling regulated by *P14AS* using KEGG pathway analysis. **(C)** TNFa factor stimulated P14AS OE cells. **(D, E)** Detection of IL6 and IL8 expression in P14AS OE cells by qRT-PCR and ELISA

In a prior study exploring putative ncRNAs encoded in the *CDKN2A/B* locus of the human genome, we screened several RNAs through a *CDKN2A*-specific RNA capture deep-sequencing approach, ultimately leading to the PCR and RNA FISH-based validation of the lncRNA *P14AS*. High levels of *P14AS* expression were detected in CC tissues, and it was able to drive the *P16-*independent proliferation of tumor cells (Ma et al. 2020). To further explore the mechanisms through which *P14AS* can regulate growth-related signaling activity, an RNA sequencing (RNA-seq) analysis was conducted comparing *P14AS*-overexpressing (P14AS OE) and control (P14AS Ctrl) cells to detect changes in gene expression as a function of *P14AS* levels. A high degree of correlation in gene expression patterns was observed when comparing two biological replicate samples, emphasizing the consistent effects of *P14AS* on patterns of gene expression (Fig. 1A). In KEGG pathway enrichment analyses exploring the functional mechanisms associated with *P14AS*, significant TNF and PI3K-Akt signaling pathway enrichment was detected among *P14AS-*related genes (FC > 1.2, *P* < 0.05) (Fig. 1B). Signaling through the TNFα-NF-κB-IL6/IL8 axis has reportedly been linked to *ANRIL*(Zhou et al. 2016). We detected *TNF/IL6/IL8* expression in P14AS OE and Ctrl cells by RT-PCR and the results showed that the expression of TNF/IL6/IL8 was elevated in the P14AS OE cells compared to the Ctrl cells (Fig. S1A). Western blotting analysis revealed that the *P14AS* OE group exhibited higher P65 protein expression than the Ctrl group (Fig. S1B). When TNF factor (100nM) was used to stimulate P14AS OE and P14AS Ctrl cells for 6 h, this resulted in the continuous upregulation of *P14AS* (Fig. 1C). Moreover, qRT-PCR and ELISA analyses demonstrated that *P14AS* overexpression was associated with higher IL6 and IL8 expression in CC cells, supporting an association between *P14AS* and NF-κB-IL6/IL8 pathway regulation (Fig. 1D-E).

### *P14AS* serves as a molecular sponge for *miR-23a-5p* and thereby regulates UBE2D3 expression

**Fig. 2:**
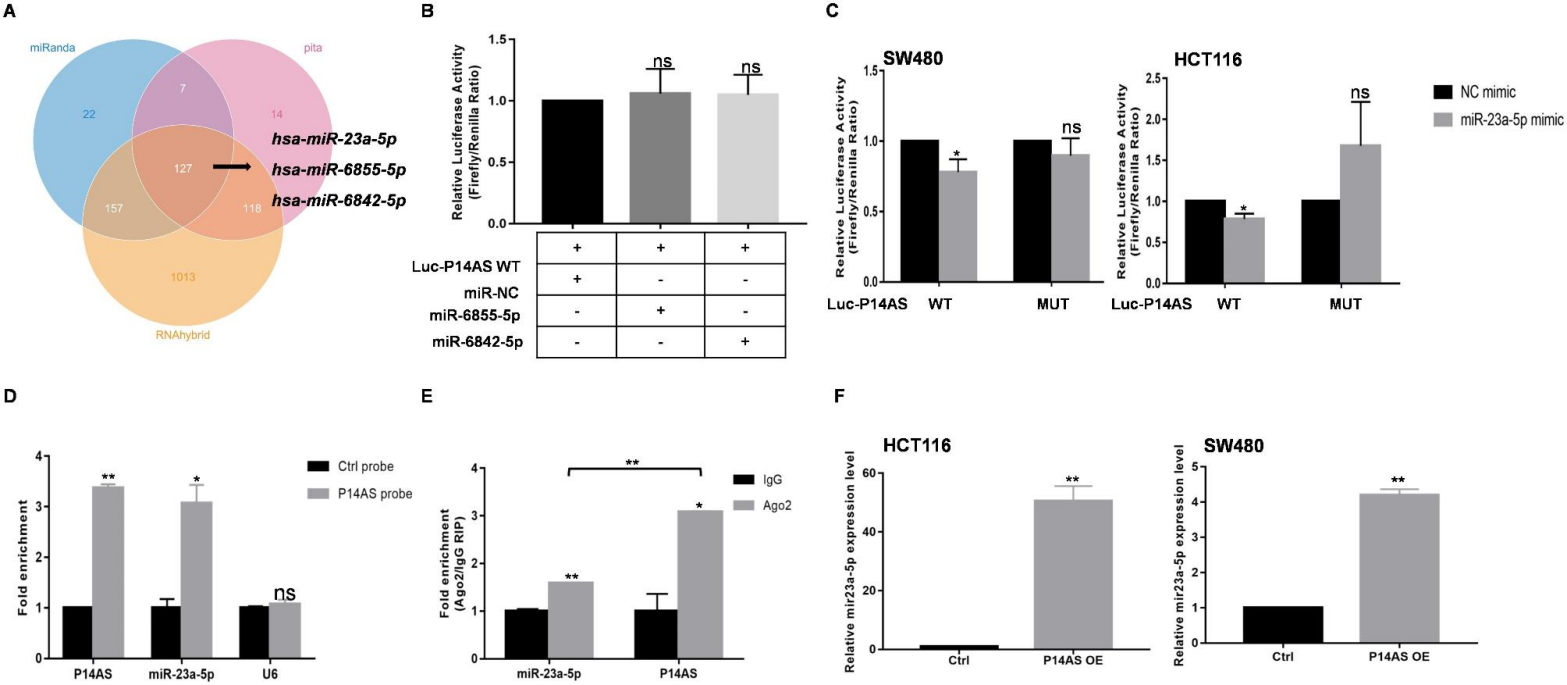
*P14AS* functions as *miR-23a-5p* sponge in CC cells. **(A)** miRanda\Pita\RNAhybrid databases predicted *P14AS* binding of miRNAs. **(B)** HCT116 cells that were co-transfected with *miR-6855-5p* or *miR-6842-5p* or control mimics and *P14AS* wild type luciferase report vector were measured for luciferase activity. **(C)** Dual-luciferase reporter assays were conducted with wild type and mutated (putative binding sites for *miR-23a-5p* was mutated) luciferase report vectors to validate *P14AS/miR-23a-5p* axis. **(D)***miR-23a-5p* was pulled down by biotin labelled *P14AS* probes. **(E)** RIP assay using AGO2 antibody found that the interaction with *P14AS* or *miR-23a-5p* and AGO2 antibody in SW480 cell. **(F)** The cells transfected with *P14AS* vector were analyzed by qRT-PCR for *miR-23a-5p* expression

To examine the mechanisms whereby *P14AS* can control the TNF-NF-κB signaling axis, the miRanda, RNA hybrid, and Pita tools were leveraged to detect overlap between *P14AS* and miRNA target genes, identifying several genes that have previously been annotated in RNA-sequencing datasets assessing the differential expression of ncRNAs. In total, 127 miRNAs were screened for interaction with *P14AS* using miRanda, RNA hybrid, and Pita databases (Table 1). Among them, only three miRNAs *miR-6855-5p*, *miR-6842-5p*, and *miR-23a-5p* were found to be associated with *P14AS*-associated proteins (Fig. 2A). To test the ability of these miRNAs to directly interact with *P14AS* in HCT116 cells, a dual-luciferase reporter assay was next conducted. While a significant reduction in luciferase activity was evident for cells co-transfected with a wild-type *P14AS* vector and a *miR-23a-5p* mimic, the same was not true following *miR-6855-5p* or *miR-6842-5p* mimic transfection. In addition, the mutation of the putative binding sequences in the *P14AS* reporter vector abrogated the ability of *miR-23a-5p* mimic transfection to suppress luciferase activity in both HCT116 and SW480 cells (Fig. 2B-C). A group of biotin-conjugated *P14AS* probes was also able to pull down *miR-23a-5p* in *P14AS* OE cells (Fig. 2D). Argonaute2 (AGO2) is a key protein member of the RNA-induced silencing complex (RISC) that controls miRNA function(Sheu-Gruttadauria et al. 2019). The use of anti-AGO2 to conduct RNA immunoprecipitation (RIP) using lysates prepared from SW480 cells resulted in *P14AS* and *miR-23a-5p* enrichment, with this enrichment being stronger for *P14AS* relative to *miR-23a-5p* (Fig. 2E). Overexpression of *P14AS* led to increased levels of the *miR-23a* precursor transcript (Table 2) and qPCR confirmed the upregulation of *miR-23a-5p* (Fig. 2F).

**Table 1:** P14AS-binding miRNAs predicted by miRanda/RNA hybrid/Pita databases

Target_miRNA	Target_miRNA	Target_miRNA	Target_miRNA
hsa-miR-3648	hsa-miR-488-3p	hsa-miR-3605-5p	hsa-miR-4722-5p
hsa-miR-8077	hsa-miR-608	hsa-miR-6769b-5p	hsa-miR-6795-5p
hsa-miR-6716-5p	hsa-miR-4728-5p	hsa-miR-203a-5p	hsa-miR-4723-5p
hsa-miR-6779-5p	hsa-miR-6076	hsa-miR-3150b-3p	hsa-miR-2392
hsa-miR-6756-5p	hsa-miR-3922-5p	hsa-miR-1233-5p	hsa-miR-1343-5p
hsa-miR-663b	hsa-miR-940	hsa-miR-6835-5p	hsa-miR-7110-5p
hsa-miR-12,119	hsa-miR-1292-5p	hsa-miR-6803-5p	hsa-miR-6785-5p
hsa-miR-939-5p	hsa-miR-4758-5p	hsa-miR-4481	hsa-miR-6797-5p
hsa-miR-6798-5p	hsa-miR-6823-5p	hsa-miR-7106-5p	hsa-miR-6886-5p
hsa-miR-8059	hsa-miR-6752-5p	hsa-miR-6090	hsa-miR-6893-5p
hsa-miR-4651	hsa-miR-6783-5p	hsa-miR-4754	hsa-miR-11181-3p
hsa-miR-6861-3p	hsa-miR-4436a	hsa-miR-4787-5p	**hsa-miR-6855-5p**
hsa-miR-619-3p	hsa-miR-664a-5p	hsa-miR-6796-5p	hsa-miR-5007-5p
hsa-miR-4706	hsa-miR-4659b-3p	hsa-miR-3691-5p	hsa-miR-4749-5p
hsa-miR-6824-5p	hsa-miR-197-5p	hsa-miR-3174	hsa-miR-10398-3p
hsa-miR-1193	hsa-miR-6742-5p	hsa-miR-135a-3p	hsa-miR-937-5p
hsa-miR-6858-5p	hsa-miR-12,115	hsa-miR-4479	hsa-miR-6880-5p
hsa-miR-6732-5p	hsa-miR-6786-5p	hsa-miR-6069	hsa-miR-23b-5p
hsa-miR-409-5p	hsa-miR-7847-3p	hsa-miR-1237-5p	hsa-miR-6505-5p
hsa-miR-3175	hsa-miR-892b	hsa-miR-6765-5p	hsa-miR-6088
hsa-miR-6751-5p	hsa-miR-328-5p	hsa-miR-3928-3p	hsa-miR-6515-5p
hsa-miR-6768-3p	hsa-miR-639	hsa-miR-765	**hsa-miR-6842-5p**
hsa-miR-6766-5p	hsa-miR-6728-5p	hsa-miR-1908-5p	hsa-miR-92a-2-5p
hsa-miR-3170	hsa-miR-3659	hsa-miR-4784	hsa-miR-7-5p
hsa-miR-6747-5p	hsa-miR-6852-5p	hsa-miR-4468	hsa-miR-638
hsa-miR-6782-5p	hsa-miR-3154	hsa-miR-6887-5p	hsa-miR-12,114
hsa-miR-6778-5p	hsa-miR-6734-5p	**hsa-miR-23a-5p**	hsa-miR-7111-5p
hsa-miR-604	hsa-miR-6081	hsa-miR-1229-5p	hsa-miR-4685-3p
hsa-miR-3188	hsa-miR-4781-5p	hsa-miR-7155-5p	
hsa-miR-4738-3p	hsa-miR-3153	hsa-miR-125b-1-3p	
hsa-miR-3190-3p	hsa-miR-4632-5p	hsa-miR-4538	
hsa-miR-6808-5p	hsa-miR-127-3p	hsa-miR-3191-3p	
hsa-miR-4745-5p	hsa-miR-6812-5p	hsa-miR-3132	

**Table 2:** The expression profiles of P14AS OE cells in RNA-seq

RNA_type	Gene	Gene_type	Ctrl	P14AS	log2(Fold_change)	*P*-value
ncRNA	SNORD3A	snoRNA	306811.1825	240601.1686	-0.350707417	0
ncRNA	RNU1-1	snRNA	2002.684525	4078.844495	1.026225328	4.26E-170
ncRNA	SNORD3B-1	snoRNA	1082.268588	275.8692032	-1.972002264	2.82E-109
ncRNA	RNU1-4	snRNA	3814.104878	2930.551201	-0.380172471	9.02E-23
ncRNA	RNVU1-7	snRNA	3373.118102	4132.930881	0.293082404	1.09E-22
ncRNA	RNU5A-1	snRNA	3443.826651	4146.583453	0.267910603	8.78E-20
ncRNA	RNY1	Y_RNA	1475.423299	1927.185697	0.385366668	3.10E-17
ncRNA	SNORD3C	snoRNA	1423.971641	1110.724801	-0.358419003	1.67E-08
ncRNA	RNU1-3	snRNA	1873.255203	1518.251528	-0.303136638	5.90E-08
ncRNA	SNORD13	snoRNA	534.0848395	709.2278189	0.409180193	7.10E-08
ncRNA	RNVU1-18	snRNA	273.124896	159.7590925	-0.773662782	1.60E-07
ncRNA	SNORD22	snoRNA	1129.215888	898.0717429	-0.330418727	3.96E-06
ncRNA	SNORD33	snoRNA	150.170981	214.3290358	0.513221255	0.000328017
ncRNA	SNORA74A	snoRNA	476.9324144	366.3426114	-0.380591319	0.000511695
ncRNA	SNORA71B	snoRNA	496.7238782	398.9898941	-0.316091895	0.003515156
ncRNA	SNORD14C	snoRNA	250.1624439	310.9023657	0.313596376	0.004446511
ncRNA	SNORA26	snoRNA	414.5350758	328.7327284	-0.334579083	0.004628115
ncRNA	RNU12	snRNA	246.568896	305.3340153	0.308397501	0.00544677
ncRNA	SNORD6	snoRNA	96.20696022	60.02505014	-0.680576568	0.005724672
ncRNA	VTRNA1-3	vault_RNA	265.8418189	324.8634643	0.289265431	0.006615095
ncRNA	RNU5D-1	snRNA	230.8808348	285.2040153	0.304845872	0.007815847
ncRNA	SNORD49A	snoRNA	368.1983591	292.339075	-0.33284052	0.007949269
ncRNA	SNORA80B	snoRNA	348.9971035	278.4071611	-0.326018743	0.011468515
**precursor_RNA**	**MIR23A**	**miRNA**	**6.160087383**	**17.49572878**	**1.505980039**	**0.015020224**
ncRNA	SNORA80A	snoRNA	242.8932457	190.8360291	-0.347988801	0.023882845
antisense	ENSG00000269968.1	antisense	17.42817375	32.52322812	0.900049059	0.025506431
ncRNA	SNORA72	snoRNA	213.7639979	166.0514396	-0.364388664	0.026179587
precursor_RNA	MIR4521	miRNA	4.684155156	13.83859564	1.562836775	0.026370783
ncRNA	RNU4ATAC	snRNA	67.39634992	92.86234509	0.462423255	0.030478252
precursor_RNA	MIR1244-3	miRNA	130.9753843	96.56743292	-0.439687063	0.034918547
ncRNA	SNORD78	snoRNA	170.2985199	130.3670142	-0.385487015	0.035411871
precursor_RNA	MIR6758	miRNA	26.07050499	12.67526834	-1.040402238	0.035432585
ncRNA	SNORD29	snoRNA	62.07394804	40.39659486	-0.619754218	0.042149632
ncRNA	SNORD14B	snoRNA	77.13983269	102.1254016	0.40479383	0.042782023

**Fig. 3:**
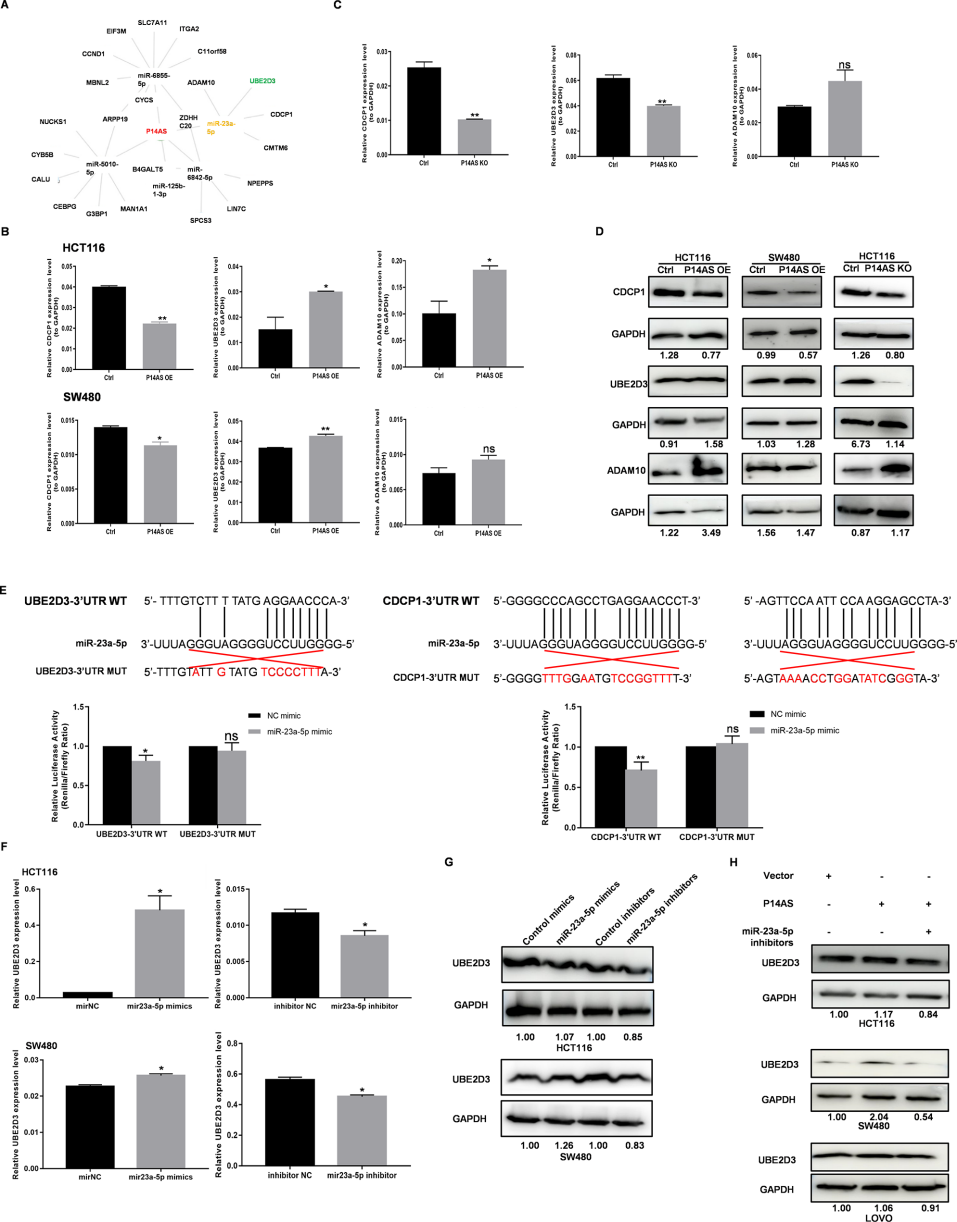
UBE2D3 is a direct target of *miR-23a-5p* in CC cells. **(A)** RNA network of *P14AS/miR-23a-5p*/target genes overlapped with TNF-NF-κB signaling pathway annotated in RNA-seq data. **(B)** P14AS OE transcriptionally changed the expression levels of *miR-23a-5p* target genes. **(C)** P14AS KO transcriptionally changed the expression levels of *miR-23a-5p* target genes. **(D)** P14AS OE or KO changed the protein levels of miR-23a-5p target genes in CC cells. **(E)***miR-23a-5p* overexpression reduced the fluorescence intensity of vector containing wild type (wt) 3’UTR of *UBE2D3* and *CDCP1*, but did not have impact on the fluorescence intensity of mutated (mut) 3’UTR of *UBE2D3* and *CDCP1*. **(F, G)***miR-23a-5p* overexpression increased the expression of *UBE2D3* and downregulation of *miR-23a-5p* reduced the expression of UBE2D3. **(H)** P14AS OE increased the protein level of UBE2D3, which was reduced by *miR-23a-5p* inhibitors in CC cells

RNA-seq data led to the identification of four target genes, including *CDCP1*, *ADAM10*, *CMTM6*, and *UBE2D3* (Fig. 3A). Of these targets, *CDCP1* is reportedly associated with PI3K/AKT signaling(Khan et al. 2021), *CMTM6* is a critical regulator of PD-L1 in a broad range of cancer cells(Burr et al. 2017), while *UBE2D3* is linked to NF-κB signaling(Chen et al. 2017) and *ADAM10* plays a role in TNF signaling(Arduise et al. 2008). Based on the results of qRT-PCR analyses, *P14AS* overexpression was found to promote both *CDCP1* downregulation and *UBE2D3* upregulation in HCT116 and SW480 cells (Fig. 3B), while *CMTM6* expression was presented in Fig. S1C. As expected, knocking out the AU-rich element (ARE) region recognized with AUF1 found in the first *P14AS* exon (P14AS KO)(Ma et al. 2020) suppressed *UBE2D3* in HCT116 cells (Fig. 3C), as further confirmed via Western blotting (Fig. 3D). The *miR-23a-5p* binding sites in the 3’-UTR regions of *CDCP1* and *UBE2D3* were next analyzed and used to construct WT and mutant *CDCP1*-3’UTR and *UBE2D3*-3’UTR luciferase reporter vectors. Co-transfection of *CDCP1*-3’UTR and *UBE2D3*-3’UTR vectors (Luc-CDCP1-3’UTR WT and Luc-UBE2D3-3’UTR WT) with *miR-23a-5p* mimics led to a reduction in luciferase activity. However, this reduction was not observed with the mutated Luc-CDCP1-3’UTR MUT and Luc-UBE2D3-3’UTR MUT constructs (Fig. 3E). Given the consistent changes in *UBE2D3* expression observed with changes in *P14AS* expression levels, it was selected as a target for further analysis. The overexpression of *miR-23a-5p* resulted in *UBE2D3* upregulation at the mRNA and protein levels in CC cells, whereas silencing *miR-23a-5p* had the opposite effect (Fig. 3F-G). Overexpressing *P14AS* promoted *UBE2D3* upregulation in these cells, while *miR-23a-5p* inhibitor transfection reversed this effect (Fig. 3H). These results thus indicate that *P14AS* can function as a *miR-23a-5p* sponge or compete with mir-23a-5p for AGO2 binding, thereby indirectly regulating *UBE2D3* expression in CC cells.

### The oncogenic activity of *P14AS* is associated with the UBE2D3-mediated degradation of IκBa and consequent NF-κB signaling activity

**Fig. 4:**
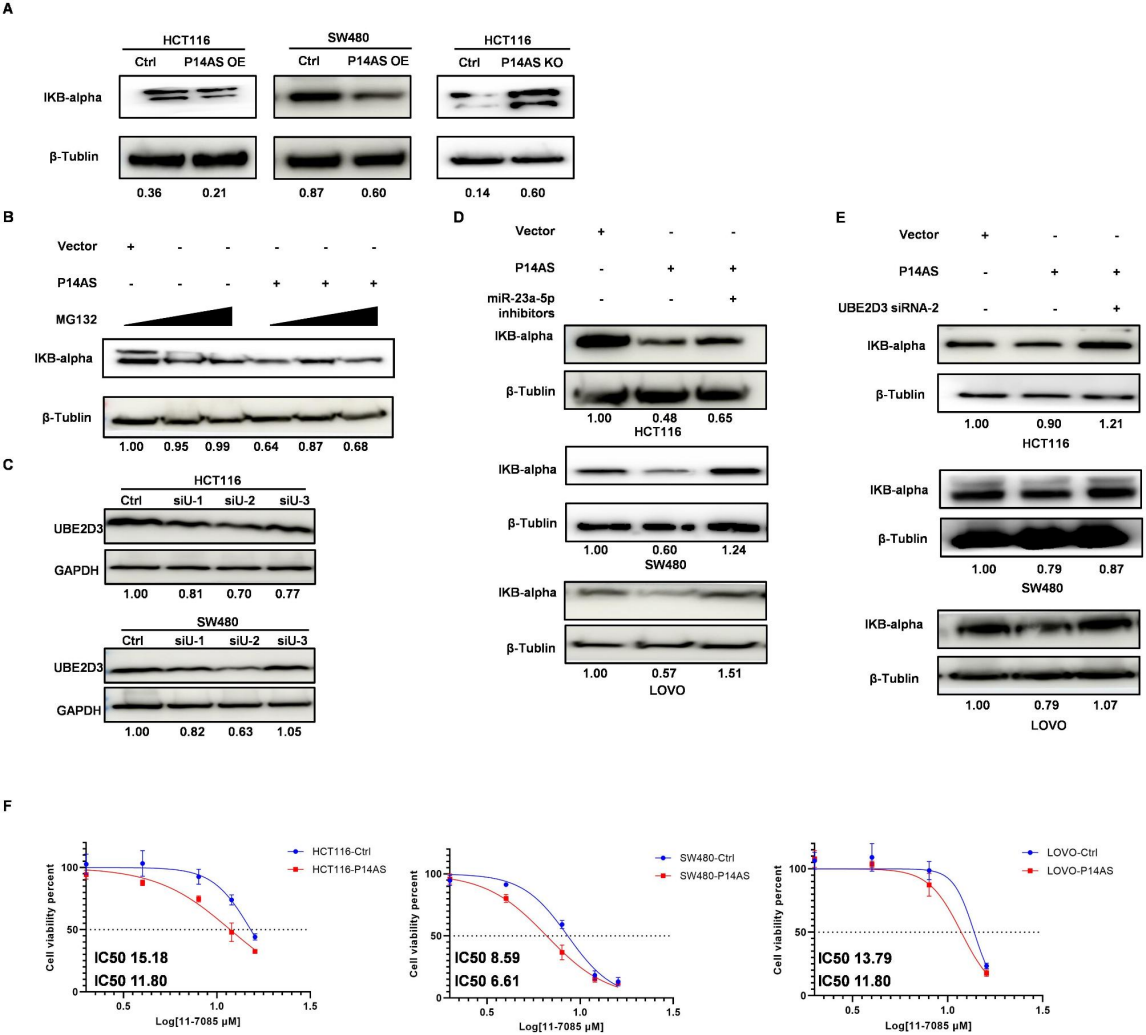
The regulatory effect of UBE2D3 on NF-κB signaling activation. **(A)** P14AS OE reduced the IκBa protein level in CC cells and P14AS KO increased the IκBa protein level in HCT116 cells. **(B)** Restoration of IκBa protein levels after treatment of P14AS OE in HCT116 cells with proteasome inhibitor MG132. **(C)** UBE2D3 protein level was knock down by siRNAs in CC cells. **(D)** P14AS OE reduced the protein level of IκBa, which was improved by miR-23a-5p inhibitors in CC cells. **(E)** P14AS OE reduced the IκBa protein level, which was improved by UBE2D3 siRNA in CC cells. **(F)** IC50 was measured for P14AS OE and Ctrl cells after BAY 11-7085 treatment

In prior reports, UbcH5c/UBE2D3 has been shown to facilitate the polyubiquitination of the inhibitory IκBa protein and its consequent proteasomal degradation, thereby enhancing NF-κB-dependent inflammation(Qi et al. 2022). UbcH5c/UBE2D3 can further mediate PCNA and histone H2A ubiquitination, thus influencing general transcriptional activity, DNA damage responses, and the replication of genomic DNA(Sakasai et al. 2023). Given these prior findings, further experiments were conducted exploring the role of UBE2D3 in CC and its associations with *P14AS* and *miR-23a-5p*. Initially, the impact of *P14AS* on signaling via a UBE2D3/IκBa/NF-κB pathway was examined through analyses of IκBa levels in P14AS OE and KO cells, with β-tubulin as a reference control. Overexpressing *P14AS* was associated with significant decreases in intracellular IκBa levels, whereas the opposite effect was observed in *P14AS* KO HCT116 cells (Fig. 4A). Notably, the treatment of HCT116 cells overexpressing *P14AS* with MG132 (a proteasome inhibitor) to inhibit proteasomal activity for 24 h resulted in the restoration of IκBa protein levels (Fig. 4B).

Subsequently, *P14AS* OE of HCT116 and SW480 cells were transfected using *miR-23a-5p* inhibitor or *UBE2D3* siRNA constructs. As expected, UBE2D3 protein levels were significantly reduced at 48 h after *UBE2D3* siRNA transfection (Fig. 4C). Western blotting additionally demonstrated the restoration of IκBa protein levels in these cells following the silencing of miR-23a-5p or UBE2D3 (Fig. 4D-E). These data were thus consistent with a model wherein *P14AS* can promote the proteasome-mediated destruction of IκBa following *UBE2D3* upregulation, thus activating NF-κB signaling activity. To further validate these findings, the established NF-κB inhibitor BAY 11-7085 (7085) was used to treat these cells, revealing lower IC50 values for this compound in P14AS OE cells together with a greater sensitivity to 7085-induced cytotoxic cell death (Fig. 4F). BAY 11-7085 inhibitor (4µM) in P14AS OE cells was used and was found to significantly reduced cell proliferation activity (Fig. S2). Further analyses thus warrant exploring the potential clinical benefits of using 7085 or other NF-κB inhibitors in CC.

**Fig. 5:**
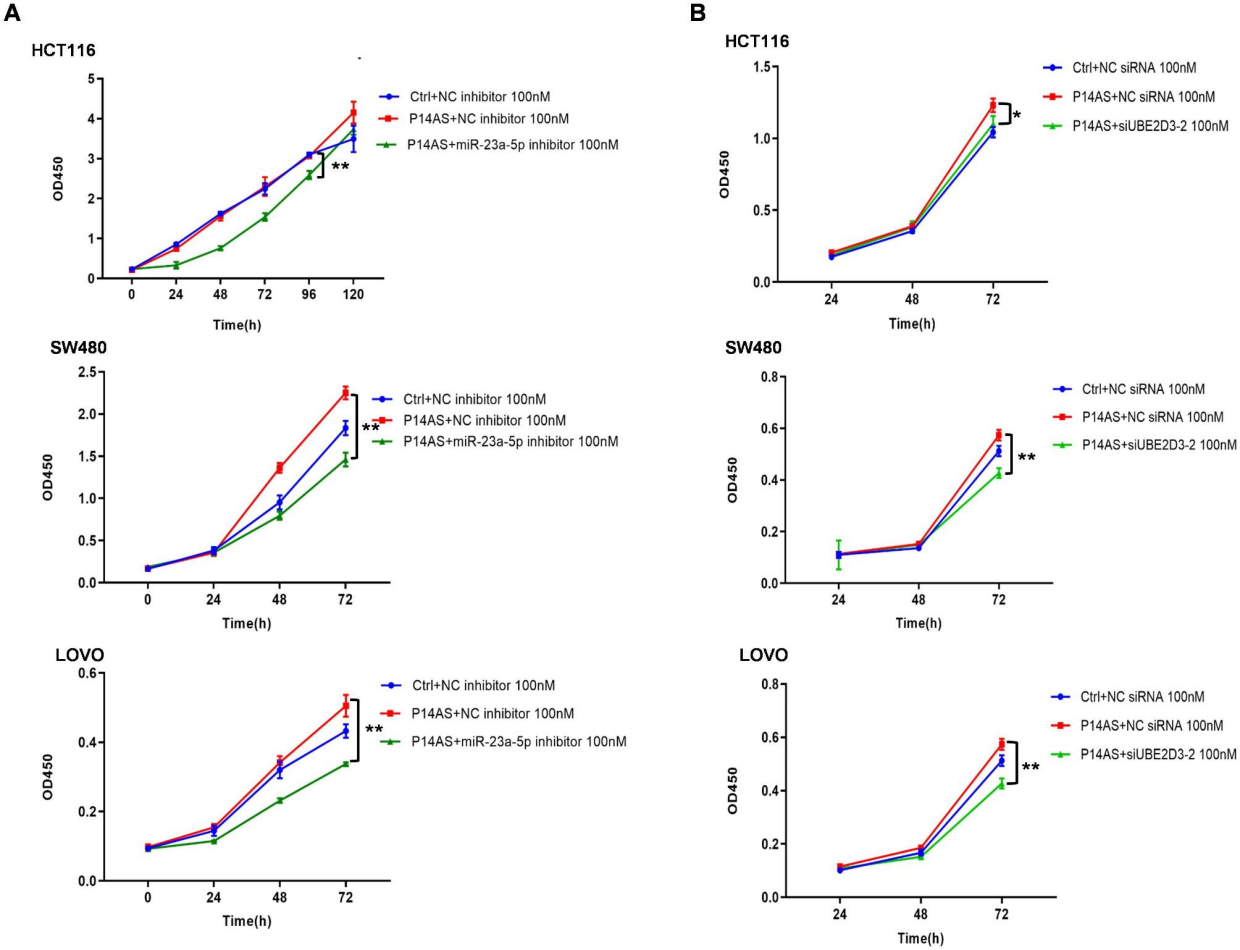
*P14AS*-*miR-23a-5p*-UBE2D3 axis is required for the CC cell growth. **(A, B)** Co-transfection with miR-23a-5p inhibitors or siUBE2D3 mitigated the carcinogenic effect of *P14AS* on cell proliferation in CC cells

*P14AS* has previously been demonstrated to promote tumorigenesis and tumor cell proliferation in NOD/SCID mice (Ma et al. 2020). To explore whether this oncogenic activity is at least partially dependent on *miR-23a-5p* and UBE2D3 in CC cells, CCK-8 assays were performed to assess cellular proliferation. This approach revealed a significant increase in proliferative activity following the overexpression of *miR-23a-5p*, while the opposite was observed when *miR-23a-5p* was knocked down (Fig. S3). Consistently, the silencing of *miR-23a-5p* eliminated the *P14AS*-associated enhancement of CC cell growth (Fig. 5A). When a siRNA construct was used to transiently knock down UBE2D3 in P14AS OE cells (Fig. S4), this UBE2D3 silencing was found to be sufficient to reverse *P14AS*-associated proliferative activity (Fig. 5B). These findings underscore that downregulation of *miR-23a-5p* or UBE2D3 can counteract the pro-proliferative effects of *P14AS* overexpression in CC cells. This supports the role of this lncRNA in promoting tumor growth and development through the UBE2D3/IκBa-mediated NF-κB regulatory axis.

### YY1 and HNF3a suppress *ANRIL* and *P14AS* transcriptional activity

**Fig. 6:**
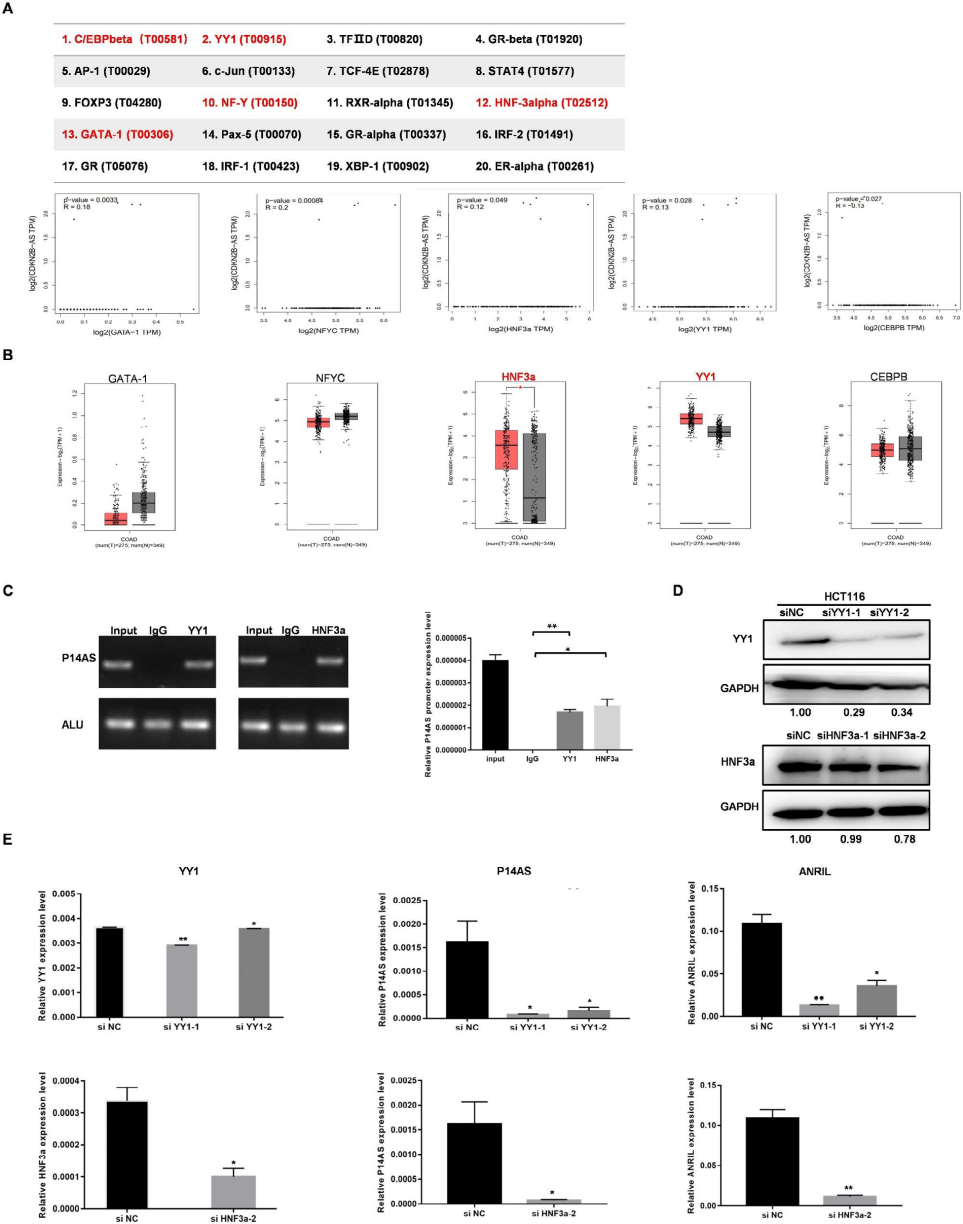
The characterization of *ANRIL* and *P14AS* promoter. **(A, B)** Analysis of transcription factors by PROMO and GEPIA databases. The orange box group represents colon adenocarcinoma (COAD) tissue and the grey box group represents normal tissue in Fig. 6B. **(C)** CHIP-PCR of the promoter region of *P14AS* by YY1 and HNF3a antibody. **(D)** YY1 and HNF3a protein level were knock down by siRNAs in HCT116 cell. **(E)** The transcript levels of *P14AS* and *ANRIL* were detected by qRT-PCR

To establish which factors are responsible for regulating *ANRIL* and *P14AS*, transcription factors predicted to bind to the 2.5 kb region upstream of these transcripts were predicted using the PROMO database (http://alggen.lsi.upc.es/cgi-bin/promo_v3/promo/promoinit.cgi?dirDB=TF_8.3). In total, 20 factors were identified as potential regulators of *P14AS* promoter activity. In general, transcription factors tend to positively regulate target gene expression. GEPIA database analyses (http://gepia.cancer-pku.cn/index.html) indicated that of these factors, YY1, GATA-1, NFYC, CEBPB, and HNF3a were correlated with the expression of *CDKN2B-AS* (*ANRIL*) (Fig. 6A). In COAD samples, GATA-1 and NFYC downregulation was observed, whereas high levels of YY1 and HNF3a expression were detected (Fig. 6B), in line with the high levels of intratumoral *P14AS* and *ANRIL* expression that was detected. We down-regulated the expression of YY1 and HNF3a in CC cells and assessed cell proliferation using the CCK8 assay. The results showed that the knockdown of the YY1 and HNF3a group significantly down-regulated the proliferative ability of the cells compared with the control group (Fig. S5). A ChIP-PCR experiment confirmed the ability of YY1 and HNF3a to bind the *P14AS* and *ANRIL* promoter region (Fig. 6C). When siRNA constructs were used to knock down the expression of YY1 and HNF3a (Fig. 6D), reductions in both *P14AS* and *ANRIL* levels were observed (Fig. 6E), confirming the ability of YY1 and HNF3a to serve as positive regulators of both *ANRIL* and *P14AS.*

### Analyses of *ANRIL* and *UBE2D3* target genes in COAD

**Fig. 7:**
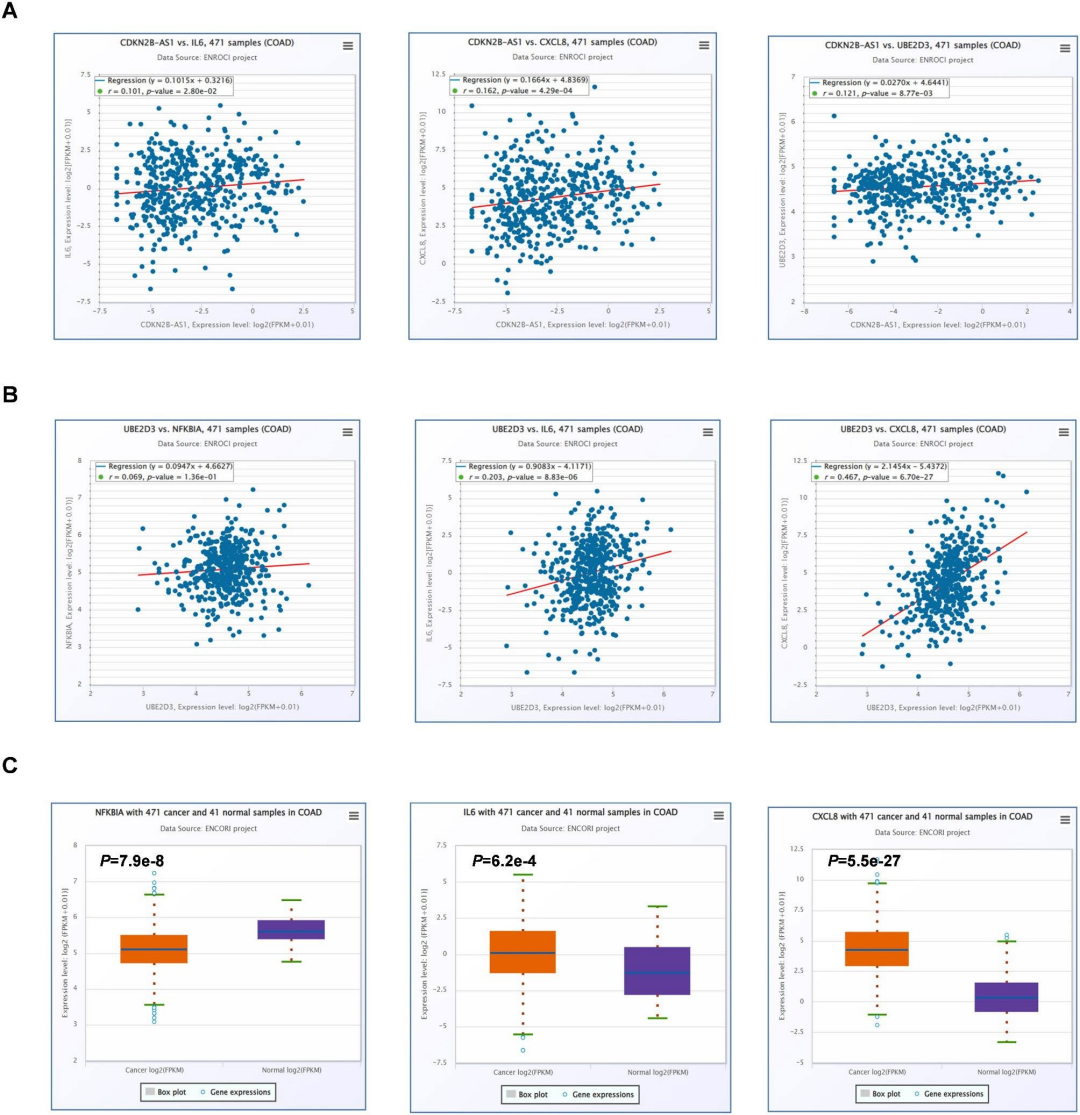
Databases analysis of *ANRIL* and its target genes in COAD. **(A)** The correlations between *CDKN2B-AS1* and *IL6*, *IL8* and *UBE2D3* by using ENCORI tool. **(B)** The correlations between *UBE2D3* and *NFKBIA, IL6* and *IL8* by using ENCORI tool. **(C)** Analysis of *NFKBIA, IL6* and *IL8* by ENCORI tool. The orange box group represents colon adenocarcinoma (COAD) tissue and the grey box group represents normal tissue

The GEPIA and ENCORI databases were next leveraged to assess correlations between the expression of *ANRIL* (*CDKN2B-AS1*) and target genes. In COAD samples, *ANRIL* expression was closely associated with *IL6*, *CXCL8 (IL8)*, and *UBE2D3* levels (Fig. 7A). A positive correlation was also detected between *UBE2D3* and the expression of both *IL6* and *IL8* at the mRNA level (*P* < 0.05), while no corresponding correlation was detected with *NFKBIA (IκBa)* expression (Fig. 7B). This suggests that UBE2D3 post-transcriptionally regulates IκBa protein levels. Our analysis of COAD tissues and normal tissues in the ENCORI database showed that *NFKBIA (IκBa)* was lowly expressed in colon cancers, and *IL6* and *IL8* were highly expressed in colon cancers (Fig. 7C), which suggests that the downstream regulatory molecules of *UBE2D3* play an important role in the development of tumorigenesis. The results indicated that while *UBE2D3* expression did not significantly differ between tumor and normal tissues, it influenced the expression of *IL6* and *IL8*, downstream factors of the NF-κB signaling pathway by regulating IκBa expression.

## Discussion

*ANRIL* is encoded in the same locus of chromosome 9 that encodes the key tumor suppressor genes *CDKN2A* and *CDKN2B*. Prior research has highlighted associations between *ANRIL* and a range of pathological conditions such as atherosclerosis, type 2 diabetes, and obesity(Razeghian-Jahromi et al. 2022). The dysregulation of *ANRIL* has also been reported in a range of cancers including pancreatic cancer(Wang et al. 2022a, b), cervical cancer(Zhao et al. 2018), breast cancer(Xu et al. 2017; Ma et al. 2022), gastric cancer(Kangarlouei et al. 2019) and multiple myeloma(Wang et al. 2020). *P14AS* was first identified as a novel hypothetical linear *ANRIL* transcript, which was previously found to promote *ANRIL* upregulation and enhance tumor cell proliferation(Ma et al. 2020). While *P14AS* is related to CC progression, the specific mechanisms linking it to oncogenic activity are not yet fully established. As such, this study was designed to clarify the molecular pathways through which *P14AS* can influence CC cell proliferation.

NF-κB signaling plays an integral role in various physiological and pathological settings, and is mediated by five transcription factor subunits that include RelA (p65), RelB, c-Rel, p105/p50, and p100/p52. Heterodimers of RelA-p50 and RelB-p52 can promote canonical and non-canonical NF-κB pathway activation. Upon phosphorylation, members of the inhibitory IκB protein family (IκBα, IκBbβ, IκBε) undergo ubiquitination and proteasomal degradation. This process frees the heterodimerized RelA-p50, allowing it to translocate into the nucleus and promote target gene upregulation(Yu et al. 2020). A growing body of research has revealed that NF-κB pathway dysregulation can contribute to the incidence of cancer and various other forms of inflammation-related disease. In liver cancer, canonical NF-κB signaling can promote inflammation and the survival of hepatocytes(He and Karin 2011), whereas this same signaling axis regulates the invasion, proliferation and migration of ovarian cancer cells(Yang et al. 2018). This pro-inflammatory canonical NF-κB signaling also acts in host defenses against injury or infection. Still, chronic infections or inflammatory activity can contribute to the incidence of certain cancers, with hepatitis B virus infection having been linked to the development of hepatocellular carcinoma (HCC). In contrast, infection with *Helicobacter pylori* is associated with an increased risk of gastric cancer, while inflammatory bowel disease is linked to an elevated risk of colorectal cancer (CRC)(Yu et al. 2020). Beyond its role in the initiation of tumorigenesis, the activation of NF-κB signaling also influences hormone-dependent breast cancer progression such that the use of an IKK inhibitor as a co-treatment can promote Akt upregulation in MCF7 cells, thereby overcoming endocrine resistance(Zhou et al. 2005). Through interactions with specific miRNAs, *ANRIL* can regulate the expression and activity of NF-κB pathway components, thereby influencing inflammatory activity and other cancer-related processes. In an ischemic stroke model system, for example, the knockdown of *ANRIL* reportedly leads to the alleviation of neuroinflammation through the *miR-671-5p*/NF-κB axis(Deng et al. 2022), whereas *circANRIL* silencing can suppress NF-κB and JNK/p38 pathway activity via promoting *miR-9* upregulation, shielding renal tubular epithelial cells against damage induced by lipopolysaccharide exposure(Deng et al. 2019). Here, *miR-23a-5p* was found to interact with the novel linear *ANRIL* transcript designated *P14AS*.

Prior evidence has suggested that *miR-23a-5p* exhibits oncogenic activity in bladder cancer(Li et al. 2018) and renal cell carcinoma(Quan et al. 2017), and it can additionally influence tumorigenesis and disease progression through interactions with specific lncRNAs or mRNAs. In glioblastoma, for example, the lncRNA*TPT1-AS1* can promote growth activity through the sequestration of *miR-23a-5p*(Gao et al. 2021), whereas the lncRNA *FLVCR1-AS1* can enhance cervical cancer cell malignancy via the *miR-23a-5p*/SLC7A11 axis(Zhou et al. 2022) and hepatoblastoma progression is at least partially driven by the SNHG9/*miR-23a-5p*/Wnt3a signaling pathway(Feng et al. 2021). Besides affecting tumor progression, *miR-23a-5p* could modulate the innate host defense by promoting mycobacteria survival and inhibiting the activation of autophagy against Mycobacterium tuberculosis (M.tb.) through TLR2/MyD88/ NF-κB pathway by targeting TLR2(Gu et al. 2017), which suggests that *mir-23a-5p* may affect the NF-κB pathway. Furthermore, we demonstrated that UBE2D3 was a direct target of *miR-23a-5p*. In contrast to the traditional model wherein miRNAs inhibited the expression of target proteins via composing RNA-induced silencing complex, *miR-23a-5p* mimics elevated the protein level of UBE2D3 in the current study. Previous studies have reported that *P14AS* upregulates the transcription of *ANRIL* by interacting with AUF1 through the ARE region of the first exon(Ma et al. 2020). The results of RT-PCR from AUF1 antibody-RIP assays showed an enrichment of UBE2D3 mRNA in the AUF1 antibody group compared to the IgG group, potentially explaining the observed increase in UBE2D3 protein level (Fig. S6). Through cooperation with the E2 ligase CDC34 and the SCF E3 ligase complex, UBE2D3 can promote IκBa polyubiquitination such that it is degraded by the proteasome, in turn activating inflammatory signaling through an NF-κB dependent pathway. The present data thus provide strong support for the existence of the *P14AS*/*miR-23a-5p*/UBE2D3/IκBa regulatory axis. By serving as a molecular sponge for *miR-23a-5p, P14AS* can promote the enhanced expression of UBE2D3 and the consequent degradation of IκBa, thereby augmenting NF-κB signaling activity at *UBE2D3* in CC cells and driving their ongoing proliferation. This enhanced NF-κB pathway activation may also contribute to a degree of chemosensitivity in these tumor cells, as levels of *ANRIL* expression have been found to correlate with tumors’ sensitivity to chemotherapeutic treatment(Zhou et al. 2021). For example, high levels of *ANRIL* expression were negatively correlated with chemotherapeutic responses in those patients with CRC undergoing treatment with a 5-FU-based regimen such that inhibiting *ANRIL* may represent a viable approach to chemosensitization(Zhang et al. 2018). Given that NF-κB can promote tumor progression in CC in the context of high *P14AS* expression levels, selectively inhibiting signaling via the canonical NF-κB pathway may represent an effective means of treating CC in the clinic. To confirm that this *P14AS*/*miR-23a-5p*/UBE2D3/IκBa axis specifically mediates NF-κB pathway activation, BAY 11-7085 was used as a selective inhibitor of IκBa phosphorylation, and IC50 values for this compound were measured in both *P14AS* OE and Ctrl cells. The P14AS OE cells exhibited a lower IC50 value relative to corresponding control cells, indicating that CC patients expressing higher levels of *P14AS* may be more susceptible to the effects of NF-κB signaling inhibitor treatment. Further studies are warranted to determine whether the NF-κB pathway is the sole downstream target of *P14AS* or if *P14AS* exerts its oncogenic effects through other molecular mechanisms.

While these results offer new mechanistic insights into the ability of *P14AS* to regulate NF-κB signaling, they are nonetheless subject to some limitations. The *P14AS*/UBE2D3/IκBa regulatory axis was validated only in cells overexpressing *P14AS.* Cells with the ARE region knocked out in the first *P14AS* exon (P14AS KO) were not used. This is because previous studies have shown that P14AS KO cells compensate by upregulating a mutated form of *P14AS*. Since the target sites for *miR-23a-5p* are present in the mutated *P14AS* sequence, P14AS KO cells could not be used in the experiments. In addition, the in vivo roles of UBE2D3 as a regulator of oncogenesis were explored by subcutaneously implanting HCT116 cells in which UBE2D3 was or was not silenced in immunodeficient mice, ultimately revealing no significant differences in tumor size or intratumoral UBE2D3 expression (Fig. S7). Further research on the oncogenic roles of *UBE2D3* is warranted. These findings suggest that *UBE2D3* may primarily drive inflammatory activity and NF-κB pathway activation related to *P14AS*’s carcinogenic effects.

## Conclusions

**Fig. 8:**
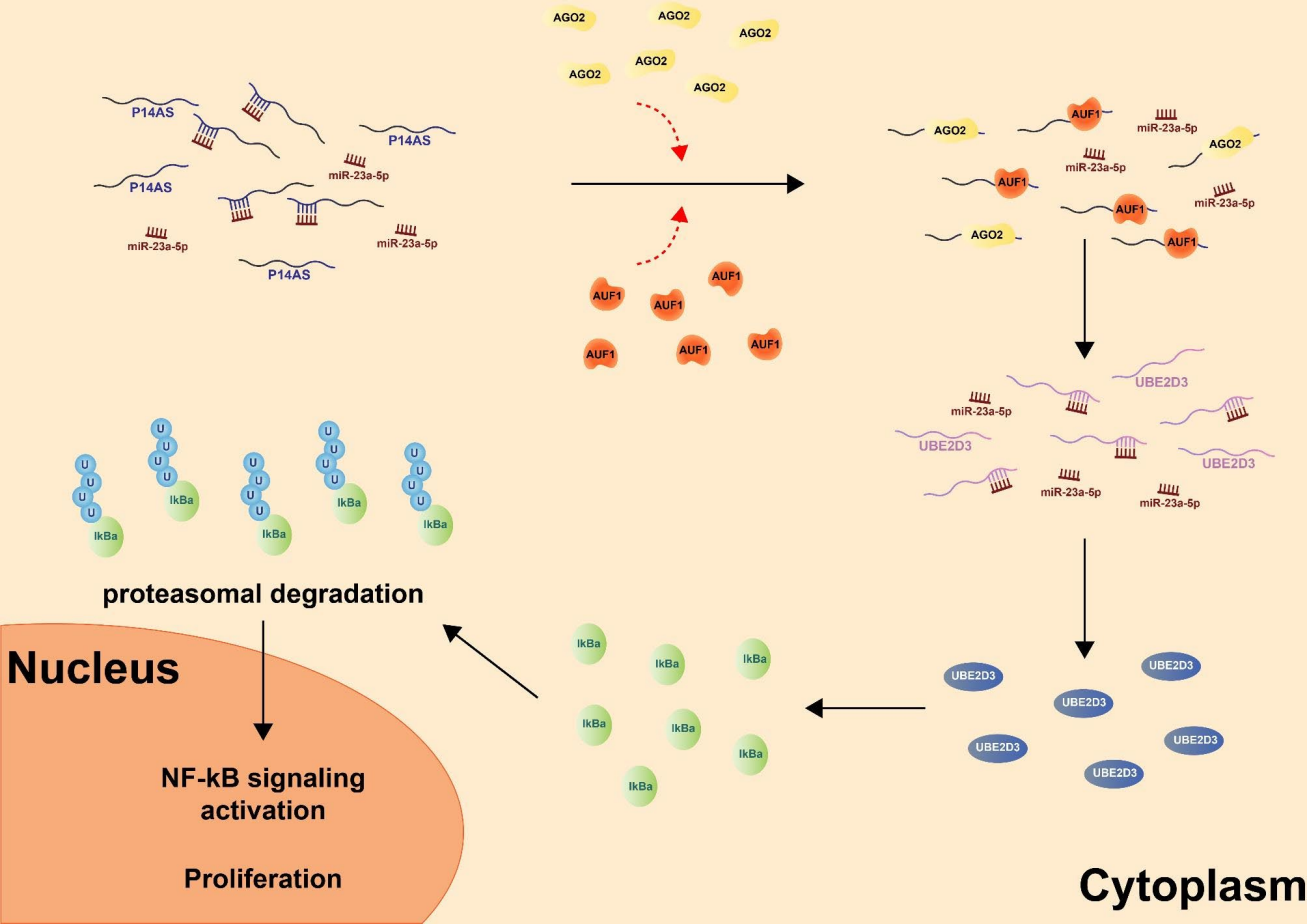
*P14AS*/*mir-23a-5p*/UBE2D3/ IκBa regulates the NF-κB signaling

The study highlights a novel association between the linear *ANRIL*-*P14AS* transcript, *miR-23a-5p*, and the UBE2D3/IκBa signaling axis in CC cells. Specifically, *P14AS* regulated NF-κB signaling activity and cellular proliferation by ultimately influencing the expression of UBE2D3 and IκBa as a direct or indirect factor (Fig. 8). Overall, these findings offer novel insight into the mechanisms that link *ANRIL* isoforms and the NF-κB pathway, potentially aiding in developing *ANRIL-*related biomarkers and therapeutic targets in patients suffering from CC.

### Electronic supplementary material

Below is the link to the electronic supplementary material.

**Supplementary Material 1**: Additional file 1 of the linear ANRIL transcript P14AS regulates the NF-κB signaling to promote colon cancer progression.

**Supplementary Material 2**: Primers and Oligos were used in this study

## Data Availability

All data generated or analyzed during this study are included in this published article [and its supplementary information files].
