# Supplementary material for: The linear ANRIL transcript P14AS regulates the NF-κB signaling to promote colon cancer progression
Source: Mol Med. 2023 Dec 1;29:162. doi: 10.1186/s10020-023-00761-z (PMC10690983; doi:10.1186/s10020-023-00761-z)
Supplement: Supplementary file 1 — Supplementary Material 1: Additional file 1 of the linear ANRIL transcript P14AS regulates the NF-κB signaling to promote colon cancer progression. [file 10020_2023_761_MOESM1_ESM.docx]

**Supplementary figure**

**
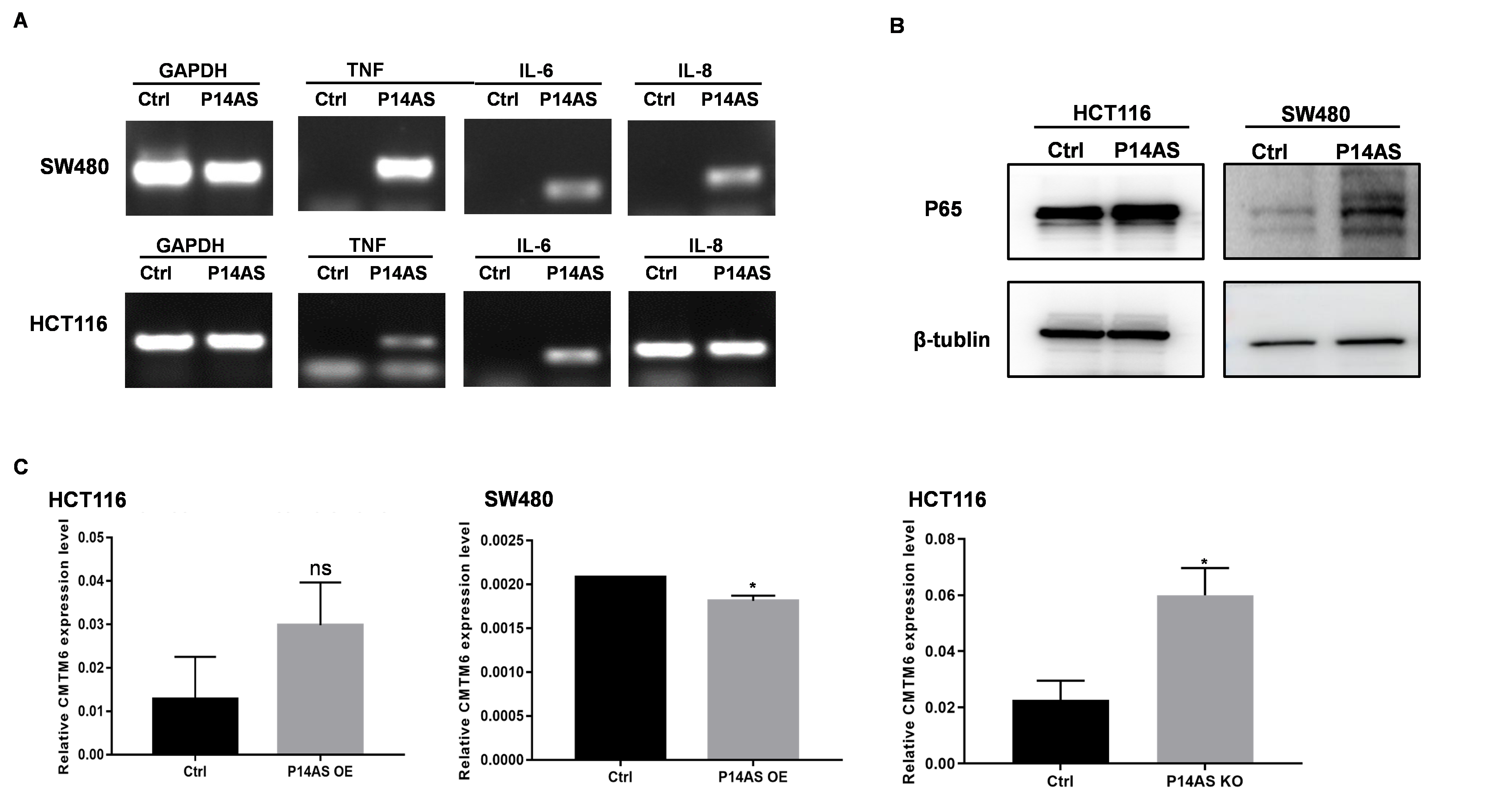
**

**Figure S1** *P14AS* regulates expression of multiple genes. (A) qRT-PCR for *TNF*/*IL6*/*IL8* expression in P14AS OE cells. (B) WB for P65 expression in P14AS OE cells. (C) qRT-PCR detection of CMTM6 expression in P14AS OE and KO cell.

**
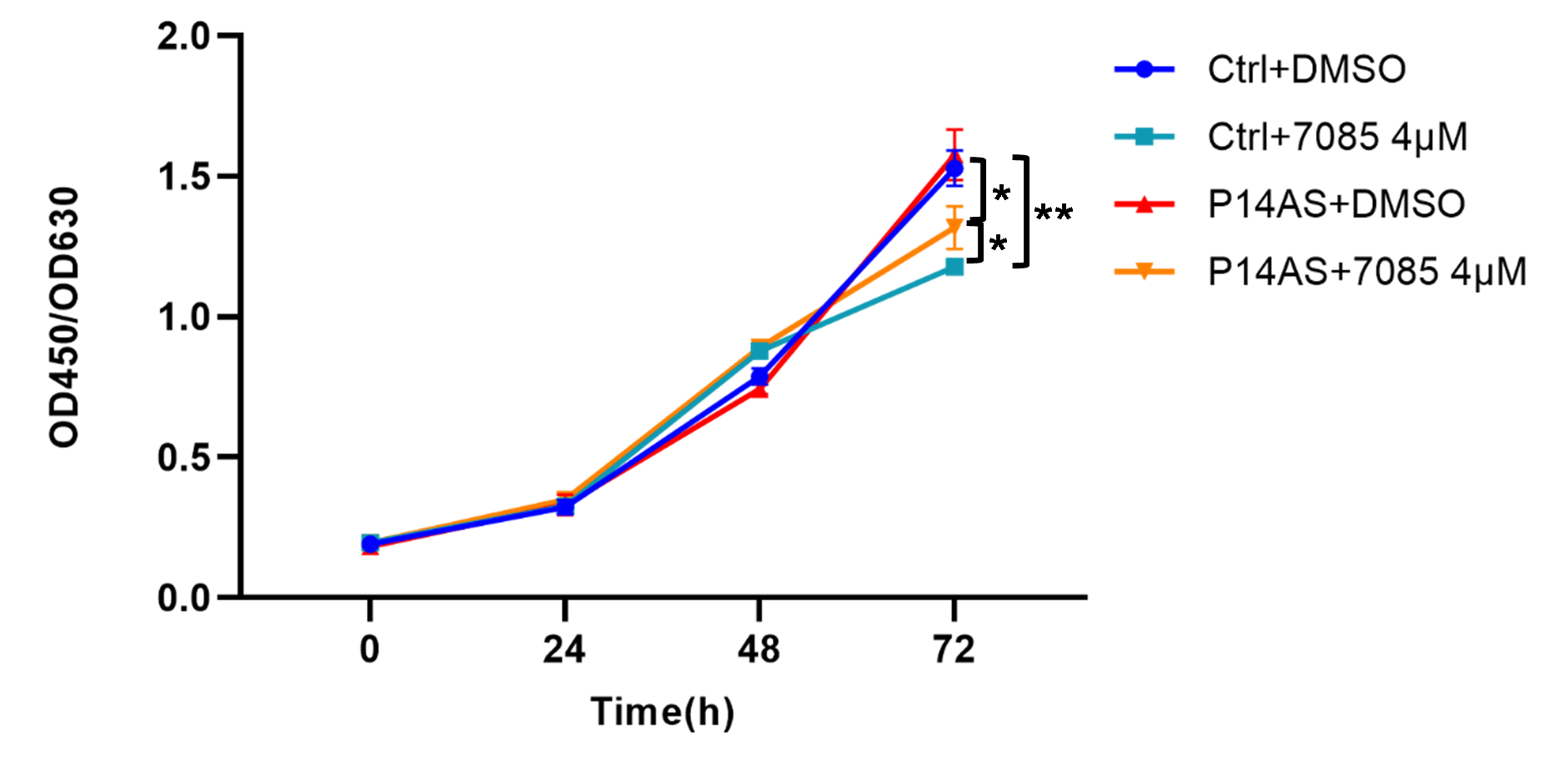
**

**Figure S2** CCK8 assays detection of cell proliferation activity in SW480 cells.


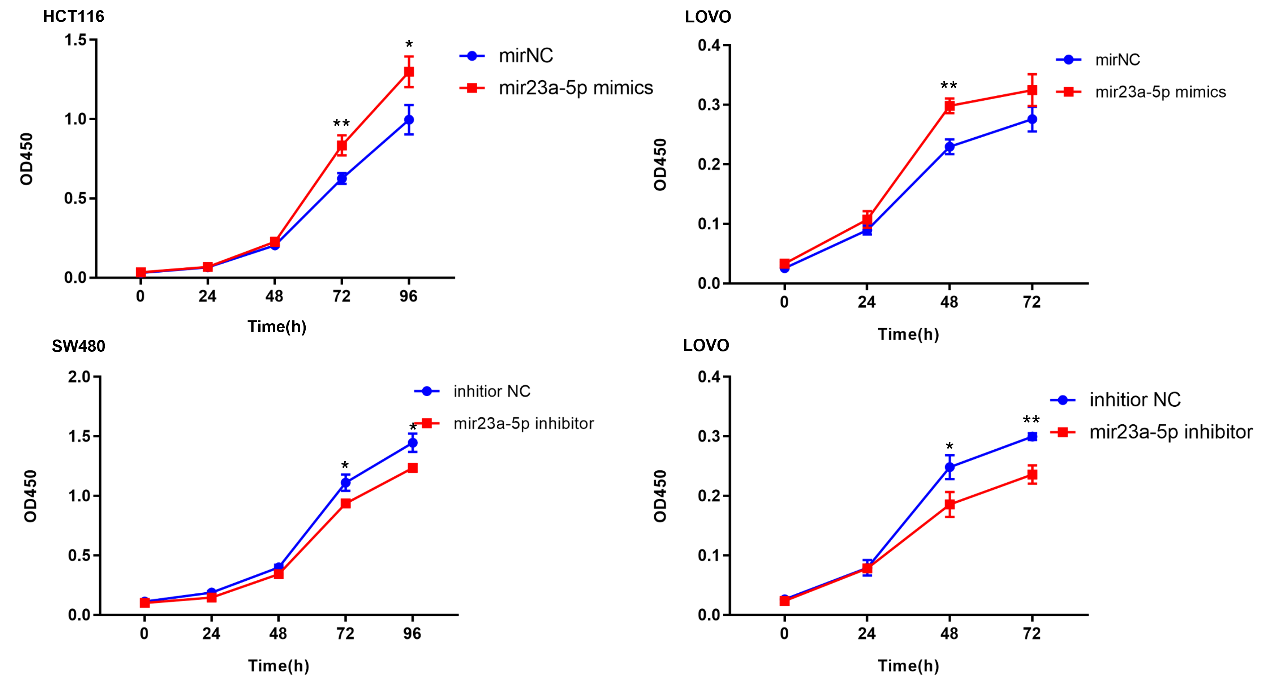


**Figure S3** CCK8 assays detection of cell proliferation activity.


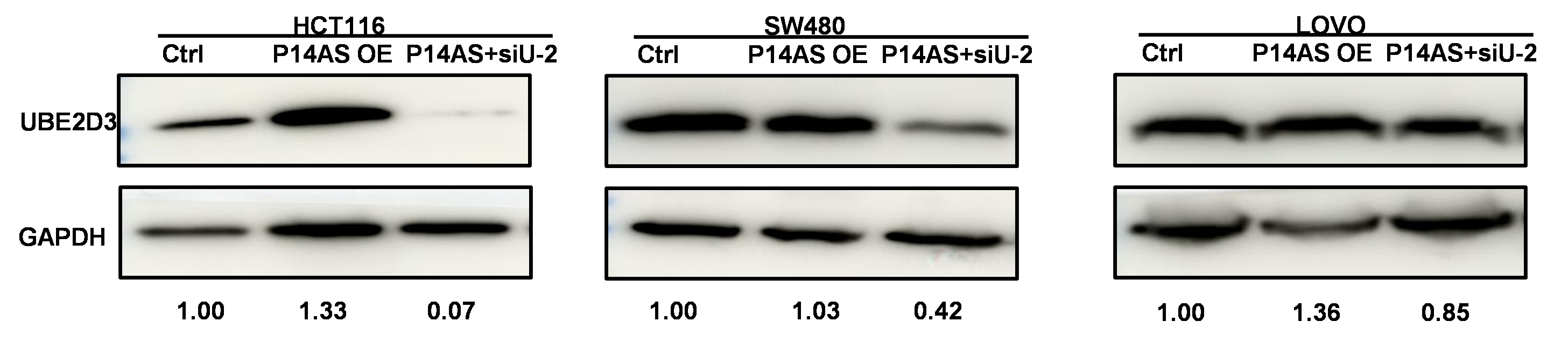


**Figure S4** Western blotting detection for UBE2D3 knockdown in P14AS OE cells.

**
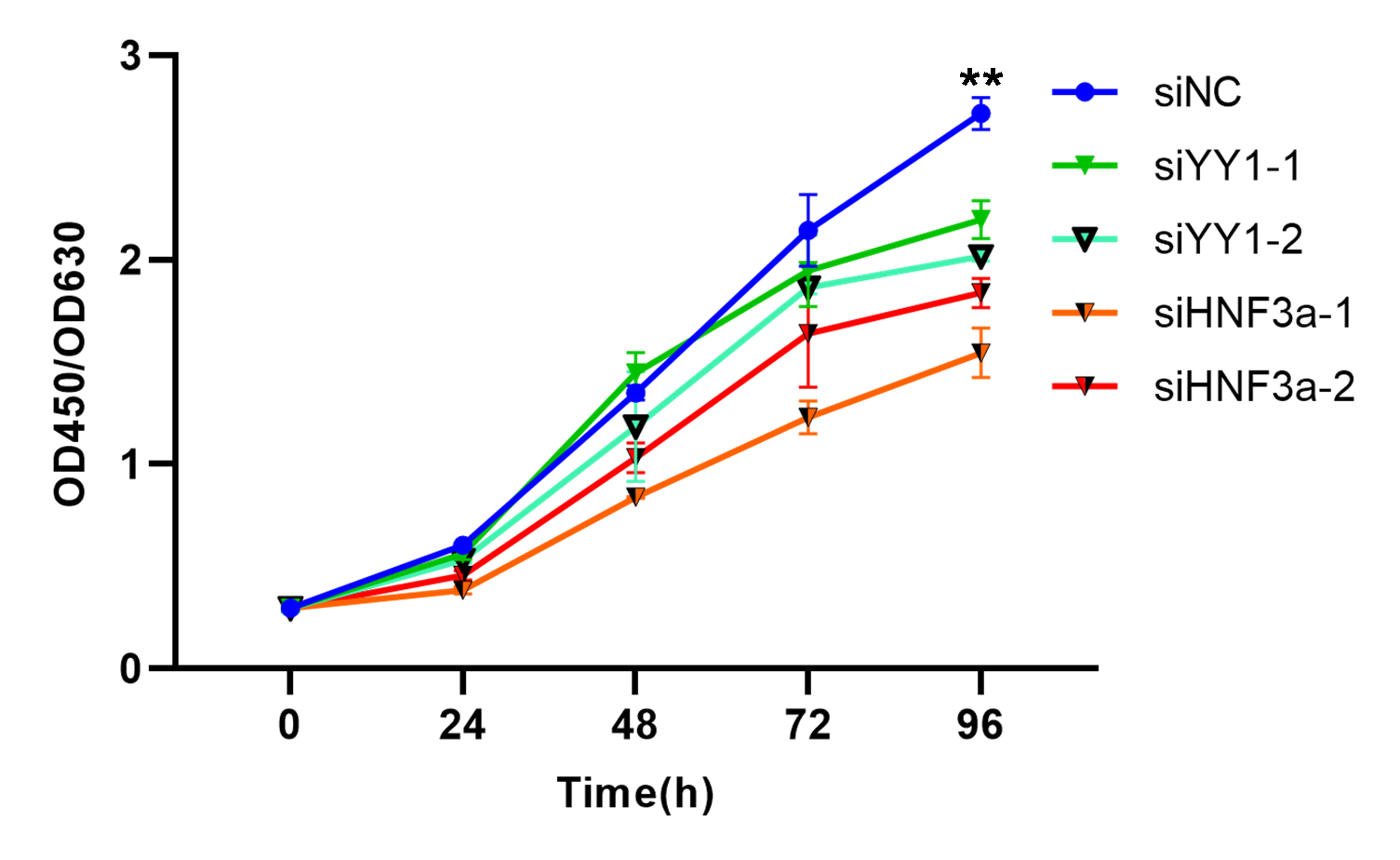
**

**Figure S5** CCK8 assays detection of cell proliferation activity in SW480 cells.


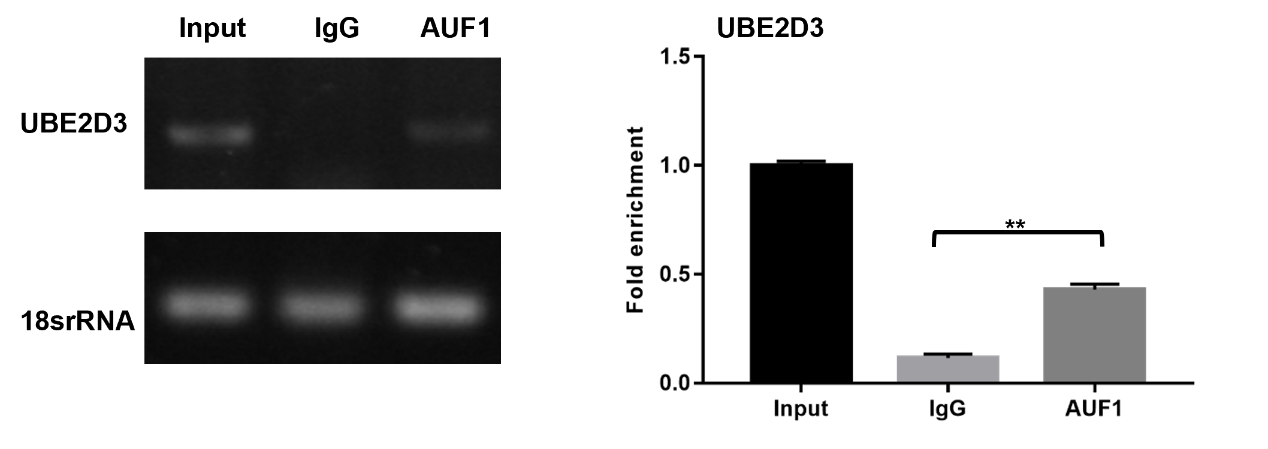


**Figure S6** RIP-PCR test of analyzing AUF1 binding RNA.


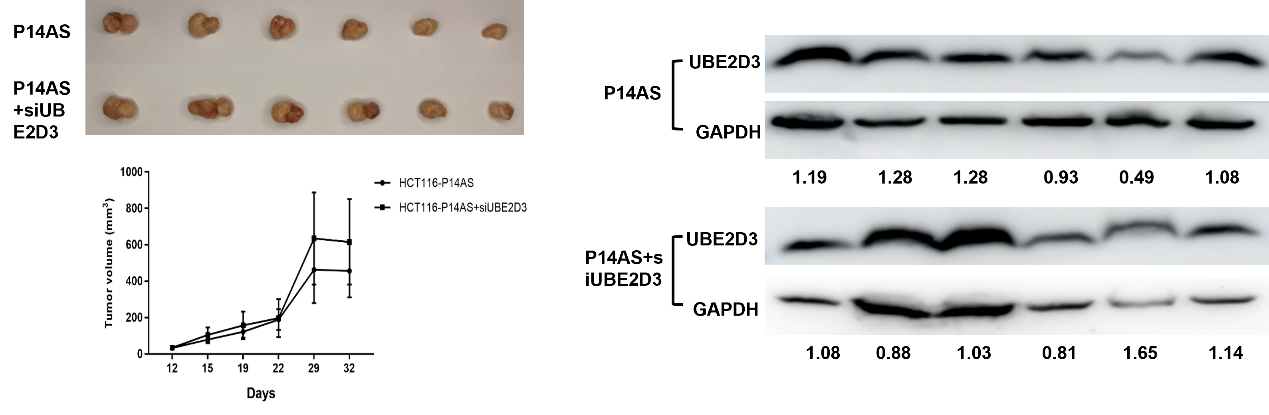


**Figure S7** UBE2D3 knockdown in *vivo* experiment.
