# Supplementary material for: The linear ANRIL transcript P14AS regulates the NF-κB signaling to promote colon cancer progression
Source: Mol Med. 2023 Dec 1;29:162. doi: 10.1186/s10020-023-00761-z (PMC10690983; doi:10.1186/s10020-023-00761-z)
Supplement: Supplementary file 2 — Supplementary Material 2: Primers and Oligos were used in this study [file 10020_2023_761_MOESM2_ESM.docx]

**Supplementary table 1**. Primers and oligos used in the study

| **experiments** | **name** | **sequence（5‘-3’）** | **size** | **annealing temperature** |
| --- | --- | --- | --- | --- |
| **qRT-PCR &** | P14AS-F | AACGGATCACATCGCTCCTG | 254bp | 58°C |
| **RIP-qPCR** | P14AS-R | TCCCCATTCGGGTTACAACG |  |  |
|  | ANRIL-F | CAGCAGAAGGTGGGCAGCAGAT | 145bp | 64°C |
|  | ANRIL-R | TTCCTCGACAGGGCAGGCAGGT |  |  |
|  | IL6-F | ACTCACCTCTTCAGAACGAATTG | 149bp | 60°C |
|  | IL6-R | CCATCTTTGGAAGGTTCAGGTTG |  |  |
|  | IL8-F | TTTTGCCAAGGAGTGCTAAAGA | 194bp | 60°C |
|  | IL8-R | AACCCTCTGCACCCAGTTTTC |  |  |
|  | TNF-F | CCTCTCTCTAATCAGCCCTCTG | 220bp | 60°C |
|  | TNF-R | GAGGACCTGGGAGTAGATGAG |  |  |
|  | RNU6-F | CGCTTCGGCAGCACATATAC | 87bp | 58°C |
|  | RNU6-R | TTCACGAATTTGCGTGTCAT |  |  |
|  | CDCP1-F | CTGAACTGCGGGGTCTCTATC | 134bp | 61°C |
|  | CDCP1-R | GTCCCCAGCTTTATGAGAACTG |  |  |
|  | ADAM10-F | TTTCAACCTACGAATGAAGAGGG | 198bp | 60°C |
|  | ADAM10-R | TAAAATGTGCCACCACGAGTC |  |  |
|  | UBE2D3-F | CCATATCAAGGCGGTGTATTCTT | 155bp | 60°C |
|  | UBE2D3-R | GGCGACCACTGTGATCTTAGA |  |  |
|  | CMTM6-F | TTTCCACACATGACAGGACTTC | 155bp | 60°C |
|  | CMTM6-R | GGCTTCAGCCCTAGTGGTAT |  |  |
|  | YY1-F | GTTCAGGGATAACTCGGCCA | 221bp | 62°C |
|  | YY1-R | CTCCGGTATGGATTCGCACA |  |  |
|  | HNF3a-F | ACAGCTACTACGCAGACACG | 70bp | 60°C |
|  | HNF3a-R | CCCAGGCCTGAGTTCATGTT |  |  |
|  | GAPDH-F | GAGATGGTGATGGGATTTC | 224bp | 62°C |
|  | GAPDH-R | GAAGGTGAAGGTCGGAGT |  |  |
|  | ALU-F | GAGGCTGAGGCAGGAGAATCG | 87bp | 60°C |
|  | ALU-R | GTCGCCCAGGCTGGAGTG |  |  |
|  | 18S rRNA-F | GCTTAATTTGACTCAACACGGGA | 69bp | 58°C |
|  | 18S rRNA-R | AGCTATCAATCTGTCAATCCTGTC |  |  |
| **ChIP-qPCR** | P14AS-promoter-F | CACTGTGCTATATCTGGAACTACAAAT | 221bp | 62°C |
|  | P14AS-promoter-R | ATTTTAAATCACATACAAAACCAAT |  |  |
| **siRNA** | siUBE2D3-1-F | CAGACAGAGAUAAGUACAATT |  |  |
|  | siUBE2D3-1-R | UUGUACUUAUCUCUGUCUGTT |  |  |
|  | siUBE2D3-2-F | GGCAGCAUUUGUCUCGAUATT |  |  |
|  | siUBE2D3-2-R | UAUCGAGACAAAUGCUGCCTT |  |  |
|  | siUBE2D3-3-F | CACUGCUAUGUGAUCCAAATT |  |  |
|  | siUBE2D3-3-R | UUUGGAUCACAUAGCAGUGTT |  |  |
|  | siYY1-1-F | CGACGACUACAUUGAACAATT |  |  |
|  | siYY1-1-R | UUGUUCAAUGUAGUCGUCGTT |  |  |
|  | siYY1-2-F | GAUGAUGCUCCAAGAACAATT |  |  |
|  | siYY1-2-R | UUGUUCUUGGAGCAUCAUCTT |  |  |
|  | siHNF3a-1-F | GCGACUGGAACAGCUACUATT |  |  |
|  | siHNF3a-1-R | UAGUAGCUGUUCCAGUCGCTT |  |  |
|  | siHNF3a-2-F | CCACUCGCUGUCCUUCAAUTT |  |  |
|  | siHNF3a-2-R | AUUGAAGGACAGCGAGUGGTT |  |  |
